# A gold-standard French-language annotated corpus of oncological entities with ICD-O normalisation

**DOI:** 10.1038/s41597-026-07583-8

**Published:** 2026-06-30

**Authors:** Johann Pignat, Milena Vucetic, Christophe Gaudet-Blavignac, Jamil Zaghir, Amandine Stettler, Fanny Amrein, Jonatan Bonjour, Jean-Philippe Goldman, Olivier Michielin, Christian Lovis, Mina Bjelogrlic

**Affiliations:** 1 Service des sciences de l’information médicale, Hôpitaux Universitaires de Genève, Geneva, Switzerland; 2 Service d’oncologie de précision, Hôpitaux Universitaires de Genève, Geneva, Switzerland

## Abstract

Developing natural language processing tools for clinical text requires annotated datasets, yet French oncology resources remain scarce. We present FRACCO (French Annotated Corpus for Clinical Oncology) an expert-annotated corpus of 1,301 synthetic French clinical cases, initially translated from the Spanish CANTEMIST corpus as part of the FRASIMED initiative. Each document is annotated with terms related to morphology, topography, and histologic differentiation, using the International Classification of Diseases for Oncology (ICD-O) as reference. An additional annotation layer captures composite expression-level normalisations that combine multiple ICD-O elements into unified clinical concepts. Annotation quality was ensured through expert review: 1,301 texts were manually annotated for entity spans by two domain experts. A total of 71,126 ICD-O normalisations were produced through a combination of automated matching and manual validation by a team of five annotators. The final dataset representing 399 unique morphology codes (from 2,290 different expressions), 273 topography codes (from 3,129 different expressions), and 3,041 unique composite expressions codes (from 10,740 different expressions). This dataset provides a reference standard for named entity recognition and concept normalisation in French oncology texts.

## Background & Summary

Most clinical information contained within electronic health records (EHR) is in unstructured, free-text format^1^. Extracting structured representations from such narrative textual data is important for both medical research and clinical practice. Natural language processing (NLP) tools, including named entity recognition (NER), are used for that purpose^2^. However, their development, evaluation and validation depend crucially on the availability of annotated datasets^3^. While significant efforts have been directed towards generating annotated datasets from English-language medical reports^4–8^, there remains a lack of high-quality, manually annotated French-language corpora^9^.

To address this, several initiatives have emerged to create French-language biomedical and clinical corpora with semantic annotations. The MERLOT corpus^10^, consists of authentic hospital reports with a specific focus on extracting medical problems, tests, and treatments, alongside contextual attributes like negation and temporality. In contrast, the QUAERO French Medical Corpus^11^ is primarily derived from biomedical literature and drug patents, focusing on ten broad UMLS semantic groups. The SIFR Annotator project^12^ has also advanced the field by providing a platform to map broad biomedical texts to several standard French ontologies (e.g., MeSH, general ICD-10). The details of these related initiatives can be found in Table 1.

**Table 1:** Comparative table of related biomedical text corpora.

Corpus	Language	Document source	Annotated Entities	Normalisation/Ontology	Size
QUAREO	French	Biomedical (MEDLINE titles, EMEA documents, EPO patents)	10 broad semantic groups (e.g., Anatomy, Chemicals/Drugs, Disorders, Procedures)	UMLS (Concept Unique Identifiers)	38 documents + 2500 publication titles 12,761 entities
MERLOT	French	Clinical (Hospital EHRs, discharge summaries)	21 Medical entities (e.g., Problems, Tests, Treatments) + 11 Attributes (e.g., Polarity, Certainty, Temporality).	Custom schema (Strong focus on temporal relations)	500 documents 44,740 entities 26,478 relations
SIFR	French	Biomedical (Agnostic / Web-based texts)	Broad biomedical concepts (Diseases, Symptoms, Procedures)	UMLS, ICD-10, MeSH, SNOMED Int.	N/A (Annotation tool & portal)
CANTEMIST	Spanish	Clinical (Synthetic oncology case reports)	Tumour morphology mentions	ICD-O-3 (Morphology only)	1,301 documents ~16,000 entities
FRASIMED	French			ICD-O-3 and SNOMED CT (Morphology only)	
FRACCO	French		Multi-attribute oncological entities (Morphology, Topography, Differentiation)	ICD-O-3 (Morphology, Topography, Differentiation and full codes)	1,301 documents 71,065 entities

Despite these efforts, resources specifically focused on oncology and utilising oncology-specific ontologies for annotation remain scarce in the French language. Recently, the translation of existing clinical corpora has emerged as a practical strategy to bypass the strict privacy regulations and high costs associated with extracting and de-identifying new clinical texts. However, fully automated annotation projection often introduces semantic drift and alignment errors, particularly in highly specialized domains. Therefore, while leveraging a translated source corpus substantially reduces the initial burden of acquiring text, it does not eliminate the need for rigorous curation.

Our work addresses this gap by building directly upon the cross-lingual texts generated by the FRASIMED^13,14^ initiative, which translated the Spanish-language CANTEMIST^15,16^ corpus to overcome initial data availability hurdles. We utilised these pre-translated synthetic texts as our foundation, applying traditional, comprehensive manual annotation to ensure gold-standard quality. The resulting French-language corpus (FRACCO) is annotated with validated International Classification of Diseases for Oncology, 3rd Edition (ICD-O-3)^17^ codes for morphology, topography, and differentiation. This resource not only complements existing corpora but also introduces expression-level normalisations, capturing complex clinical expressions that combine multiple ICD-O-3 components. By presenting this dataset, we aim to support the development and evaluation of NLP applications in oncology, particularly for French-language clinical data, and to facilitate cross-lingual research

The dataset includes 71,126 total entity annotations, divided into four categories aligned with ICD-O-3 labels (Table 2): *morphologie* (the histological type and biological behaviour of the neoplasm), *topographie* (the anatomical site of the neoplasm), *differenciation* (the histological grade of the neoplasm), and a high-level composite label, *expression_CIM*, which groups the multiple ICD-O-3 components into a single unified clinical expression. Each annotated entity was strictly normalised using the ICD-O-3 terminology; atomic entities were assigned exact codes, while expression-level annotations received composite codes combining the morphology, topography, and differentiation axes.

**Table 2:** Overview of the Dataset.

Category	Total Annotations	Unique ICD-O-3 codes	Unique Text Strings
*morphologie*	25,634	399	2,290
*topographie*	18,805	273	3,129
*différenciation*	1’053	5	181
*expression_CIM*	25,634	3,041	10,740
Total	71’126	-	14,962

To illustrate the clinical concepts most represented in the corpus and offer insight into its ontological coverage, the 15 most frequently occurring ICD-O-3 codes for *morphologie*, *topographie*, and the composite *expression_CIM* are presented in Tables 3, 4, and 5, respectively. By aggregating these distributions by their normalised codes rather than their raw text spans, these tables resolve lexical scattering and provide a direct view of the dataset’s core clinical distribution.

**Table 3:** Top 15 Codes for morphologie (out of 399 unique codes).

Rank	ICD-O-3 Code	Most Common Expression	Count	%	Cumulative %
1	8000/1	lésion	10545	41.14%	41.14%
2	8000/6	métastases	4321	16.86%	57.99%
3	8000/9	infiltration	3054	11.91%	69.91%
4	8000/3	cancer	1642	6.41%	76.31%
5	8140/3	adénocarcinome	893	3.48%	79.80%
6	8010/3	carcinome	501	1.95%	81.75%
7	8500/3	carcinome canalaire infiltrant	341	1.33%	83.08%
8	8720/3	mélanome	254	0.99%	84.07%
9	8070/3	carcinome épidermoïde	199	0.78%	84.85%
10	8246/3	tumeur neuroendocrine	154	0.60%	85.45%
11	8310/3	carcinome à cellules claires	145	0.57%	86.01%
12	8010/9	carcinomatose	135	0.53%	86.54%
13	8041/3	carcinome à petites cellules	129	0.50%	87.04%
14	8800/3	sarcome	81	0.32%	87.36%
15	9590/3	lymphome	64	0.25%	87.61%

**Table 4:** Top 15 codes for topographie (out of 273 unique codes).

Rank	ICD-O-3 Code	Most Common Expression	Count	%	Cumulative %
1	C34.9	pulmonaire	2038	10.84%	10.84%
2	C22.0	hépatiques	1287	6.84%	17.68%
3	C76.1	axillaire	989	5.26%	22.94%
4	C50.9	sein	942	5.01%	27.95%
5	C76.0	sus-claviculaire	761	4.05%	32.00%
6	C41.9	osseuse	725	3.86%	35.85%
7	C49.9	tissus mous	499	2.65%	38.51%
8	C76.3	pelvienne	435	2.31%	40.82%
9	C38.3	médiastinale	422	2.24%	43.06%
10	C34.1	lsd	397	2.11%	45.17%
11	C64.9	rénal	389	2.07%	47.24%
12	C48.0	rétropéritonéale	381	2.03%	49.27%
13	C77.9	ganglionnaire	378	2.01%	51.28%
14	C48.2	péritonéale	331	1.76%	53.04%
15	C76.2	abdominale	323	1.72%	54.76%

**Table 5:** Top 15 codes for expression_CIM (out of 3,041 unique code combinations).

Rank	ICD-O-3 Code	Most Common Expression	Count	%	Cumulative %
1	8000/1	lésion	2954	11.52%	11.52%
2	8000/6	métastase	1734	6.76%	18.29%
3	8000/9	récidive	1197	4.67%	22.96%
4	8000/3	tumeur primaire	662	2.58%	25.54%
5	C34.9 8000/1	nodules pulmonaires	641	2.50%	28.04%
6	C22.0 8000/1	lésions hépatiques	506	1.97%	30.01%
7	C77.9 8000/1	adénopathies	484	1.89%	31.90%
8	C77.1 8000/1	adénopathies médiastinales	463	1.81%	33.71%
9	C22.0 8000/6	métastases hépatiques	303	1.18%	34.89%
10	C77.0 8000/1	adénopathie sus-claviculaire	287	1.12%	36.01%
11	C34.1 8000/1	masse dans le lobe supérieur	275	1.07%	37.08%
12	C50.9 8000/1	néoplasie mammaire	268	1.05%	38.13%
13	C77.2 8000/1	adénopathies rétropéritonéales	260	1.01%	39.14%
14	C34.9 8140/3	adénocarcinome pulmonaire	220	0.86%	40.00%
15	C34.9 8000/6	métastases pulmonaires	205	0.80%	40.80%

## Methods

### Source text corpus and translation

**Fig. 1:**
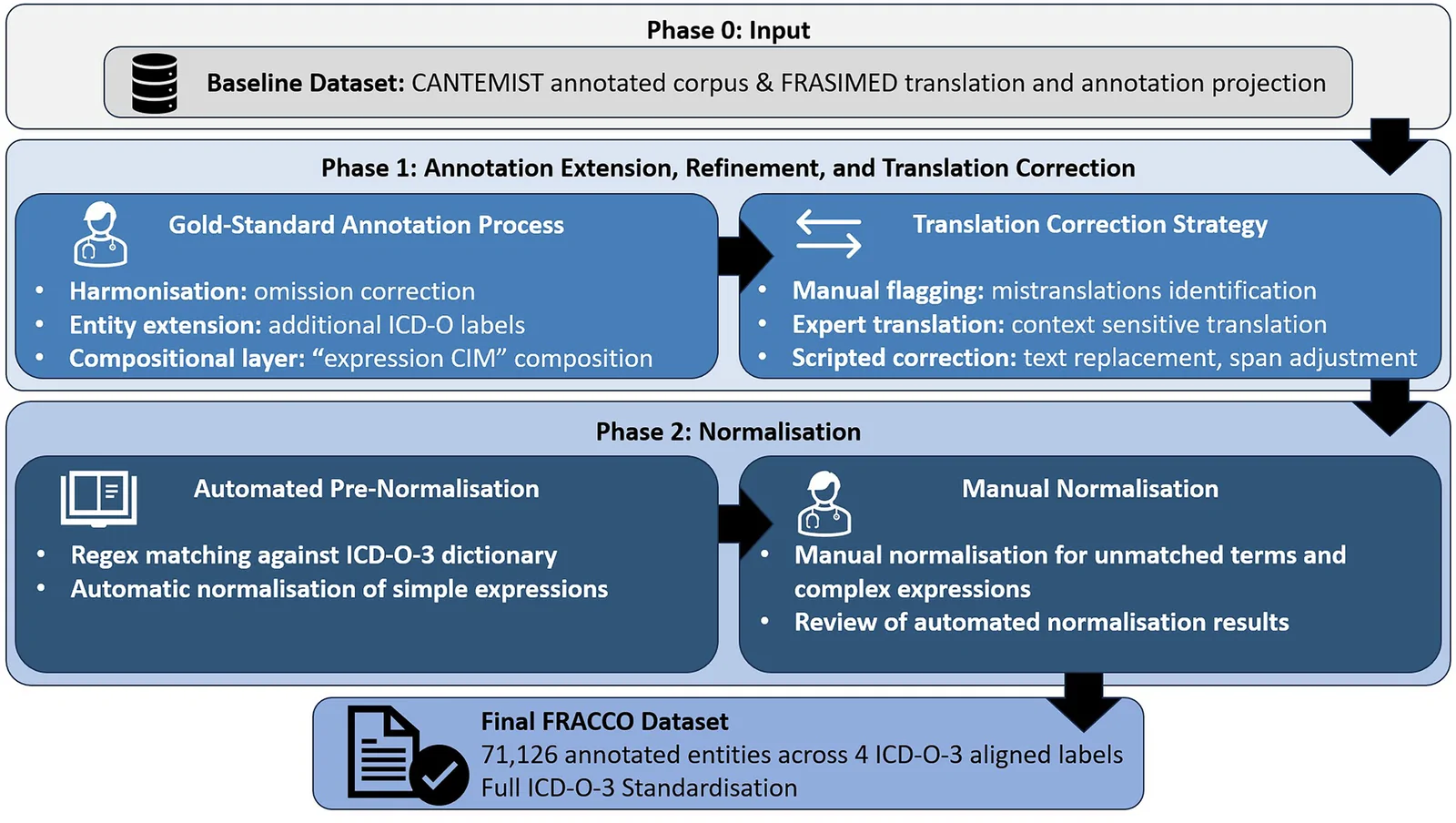
Overview of the FRACCO dataset annotation refinement and normalisation pipeline. The flowchart illustrates the sequential methodology used to generate the final gold-standard corpus from the baseline CANTEMIST and FRASIMED datasets (Phase 0). The process involves parallel manual annotation and translation correction workflows (Phase 1), followed by a hybrid automated and manual ICD-O-3 concept normalisation (Phase 2).

The FRACCO^18^ corpus presented in this dataset (the full creation pipeline of which is outlined in Fig. 1) originates from the CANTEMIST corpus, a publicly available resource developed as part of the IberLEF 2020 challenge for named entity recognition and normalisation in oncology-related Spanish clinical texts. CANTEMIST consists of de-identified, synthetic clinical case reports focused on cancer diagnoses and terminology, annotated with morphology codes from the ICD-O-3.

To facilitate multilingual research and extend the usability of the CANTEMIST corpus to French-speaking contexts, the texts were translated from Spanish to French as part of the FRASIMED project. FRASIMED is a parallel corpus of synthetic clinical documents designed to support the development of clinical NLP tools across languages. The translation process was conducted using document-level machine translation via DeepL Pro (DeepL SE, Cologne, Germany; https://www.deepl.com).

The translated French texts served as the foundation for the present dataset. The CANTEMIST subset of 1,300 documents from FRASIMED was selected to reflect a broad range of oncological expressions and ICD-O-3 morphological entities present in the original corpus.

### Annotation projection

The original Spanish CANTEMIST corpus contained manual annotations of oncology-related entities, specifically morphological terms, encoded with ICD-O-3 morphology codes. These annotations were produced using the BRAT annotation tool (https://brat.nlplab.org)^19^, which employs a standoff format where entity mentions and associated metadata (such as normalisation codes) are stored in.ann files alongside the raw text in.txt format.

To preserve the annotated structure, the original CANTEMIST annotations were automatically projected onto the French translations. Because translation inherently alters document length and character indices, this projection was performed using the FRASIMED alignment pipeline to recalculate exact entity positions. First, the source and target texts were segmented to establish a strict one-to-one parallel alignment at the sentence level. Next, a secondary model performed word-level alignments between the corresponding Spanish and French sentences based on cosine similarity, which effectively accounted for complex translation shifts such as one-to-many and many-to-one token relationships. To define the precise entity boundaries in French, a greedy span-matching strategy was applied: if two discontinuous tokens were aligned, the entire text span between them, including any unaligned intermediate tokens, was extracted as a single continuous entity. Finally, based on this updated token mapping, the new global start and end character offsets were calculated to produce the updated.ann files. Since the texts were synthetic and structurally aligned, this method allowed for a relatively accurate transfer of annotations from the source to the target language.

However, due to lexical and syntactic differences between Spanish and French, a portion of the annotations required manual verification. In cases where phrase boundaries shifted during translation or where medical terms were rephrased, the projected annotations were reviewed and adjusted to ensure correct span alignment and semantic equivalence. The projected annotations thus served as a baseline for the extended annotation work conducted in the subsequent phases of the dataset construction.

### Refinement and extension

Following the initial projection of annotations onto the French-translated corpus, substantial effort was invested in this project to refine and extend the annotation layer to improve coverage, consistency, and semantic depth. While the original CANTEMIST annotations provided a strong foundation, they were limited in scope, covering only morphological oncology terms, and contained several inconsistencies, including missing entity mentions and incomplete normalisations.

Manual reannotation of the projected dataset addressed these shortcomings with a gold-standard quality. Annotators reviewed all documents to correct span alignment errors introduced during translation, and to recover relevant oncology terms that had been omitted in the original Spanish annotations. This process also involved harmonising entity boundaries and ensuring adherence to consistent annotation guidelines.

In addition to correcting and expanding on existing annotations, two new categories of ICD-O-3 entities were introduced: topographical codes (describing the anatomical site of the tumour) and histology differentiation codes (indicating tumour grading). These were annotated de novo across the entire corpus using ICD-O-3 standards, substantially enriching the dataset’s expressiveness.

**Fig. 2:**
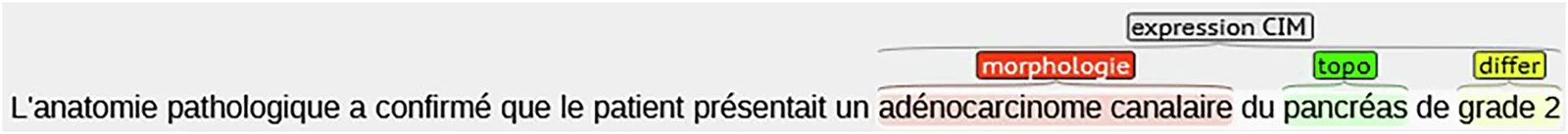
Example of a composite clinical entity annotation. A French clinical sentence annotated within the BRAT interface. Individual text spans are tagged by entity type (red: morphologie, green: topo [topography], yellow: differ [differentiation]). These atomic entities are linked under a unified compositional layer (grey: expression CIM).

Crucially, a new complete expression-level layer, termed “expression_CIM”, was added to normalise complex expressions involving combinations of morphology, topography, and differentiation into unified entities. It should be noted that the relationship between an expression_CIM annotation and its atomic components is not defined by explicit relational links in the standoff format. Instead, it is represented implicitly through the spatial overlap of their text spans; the expression_CIM acts as an overarching, standalone entity that encapsulates the components of the complex clinical concept. A visual representation of this schema within the BRAT interface is provided in Fig. 2. To enrich this example, the corresponding standoff annotation data, including precise character spans and exact ICD-O-3 normalisations for both atomic and composite entities, is detailed in Table 6.

**Table 6:** BRAT data relative to annotation example of Fig. 2.

Entity Type	Text Span	Character Span	ICD-O-3 Code
*morphologie*	adénocarcinome canalaire	65-89	8500/3
*topographie*	pancréas	94-102	C25.9
*différenciation*	grade 2	107-114	2
*expression_CIM*	adénocarcinome canalaire du pancréas de grade 2	65-114	C25.9 8500/32

**Fig. 3:**
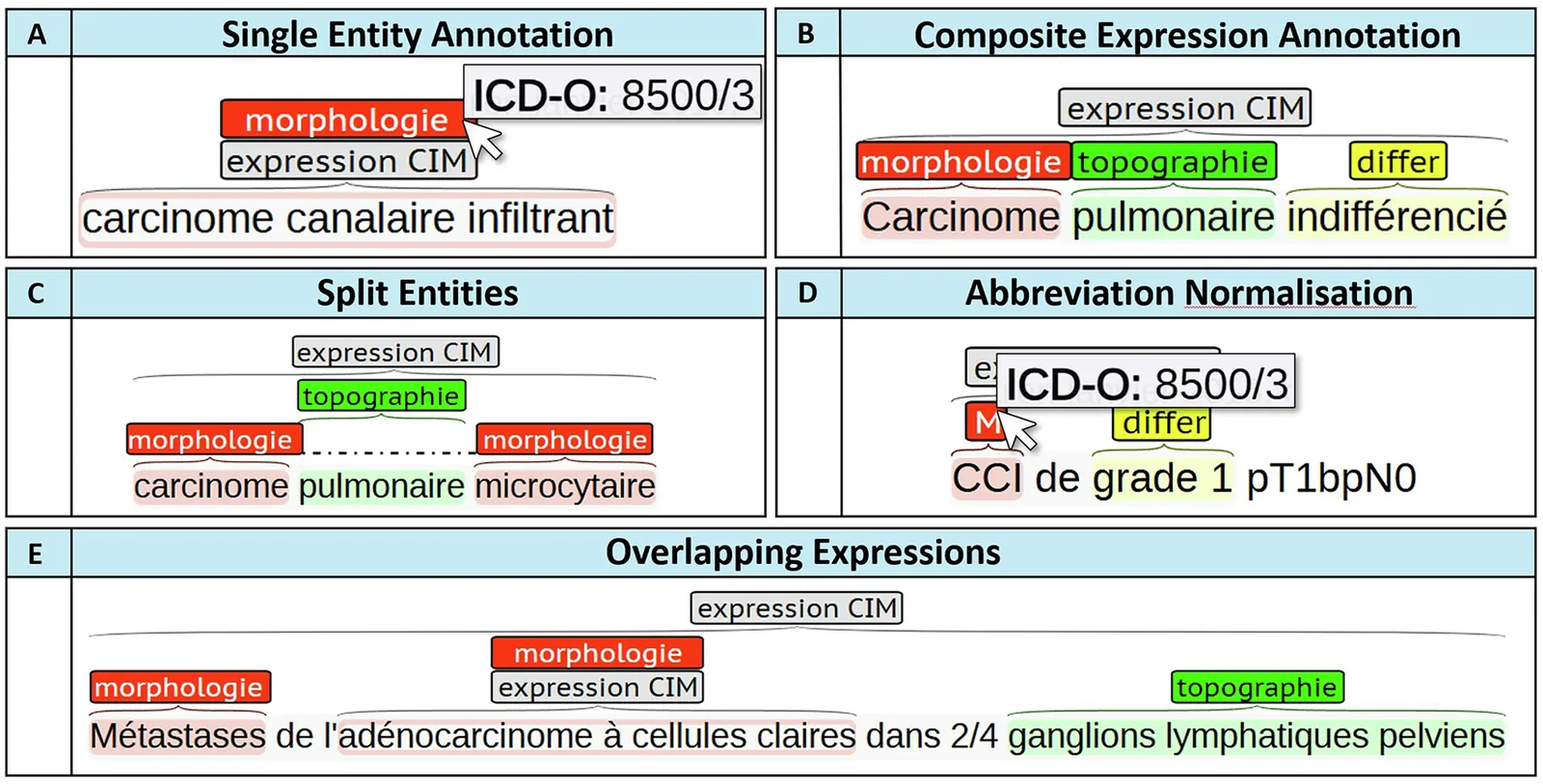
Examples of the BRAT annotation schema applied to French clinical text. (**A**) Single entity normalisation. (**B**) Composite expression combining morphology, topography, and differentiation. (**C**) Split entity annotation for discontinuous text. (**D**) Normalisation of clinical abbreviations. (**E**) Handling of complex overlapping expressions. Colored labels indicate the specific entity type (red: morphology, green: topography, yellow: differentiation), while grey boxes (expression CIM) denote the compositional layer linked to an ICD-O-3 code.

These annotations capture clinically meaningful multi-attribute expressions (e.g., “carcinome épidermoïde bien différencié du poumon”) and are each linked to their corresponding ICD-O-3 codes. The creation of this layer enables higher-level semantic analysis and facilitates use cases such as relation extraction and document classification. To illustrate the linguistic and structural complexities that necessitated this rigorous manual review, Fig. 3 presents a gallery of representative annotation scenarios from standard single-entity mappings to the resolution of non-contiguous composite expressions and ambiguous clinical abbreviations.

As a result of this extensive refinement and extension process, the total number of annotations in the corpus grew from approximately 16,000 in the original CANTEMIST dataset to over 70,000 in the current version. These annotations represent 2,290 unique *morphologie* expressions, 3,129 unique *topographie* expressions, 181 unique *différenciation* unique expressions, and 10,740 unique *expression_CIM* expressions. These unique expressions were mapped to a total of 399 ICD-O-3 morphology codes, 273 topography codes, all four differentiation codes, and 3,041 unique expression_CIM after normalisation. This expanded and corrected annotation layer provides a resource for French-language biomedical NLP and cancer information extraction tasks.

### ICD-O-3 normalisation

To ensure semantic consistency and interoperability with standardised cancer ontologies, all entity annotations were normalised using the ICD-O-3. The normalisation process combined automated matching with manual review and was applied to both individual entity annotations (*morphologie*, *topographie*, *différenciation*) and higher-level composite expressions (*expression_CIM*).

For single-label terms and simple expressions, a dictionary-based matching approach was used. Each annotated span was compared via regular expressions to entries in a comprehensive ICD-O-3 terminology lexicon. Exact matches were assigned the corresponding ICD-O-3 code. When a term or expression already had a code from the original CANTEMIST annotations, the two codes were compared. Discrepancies between the dictionary-based match and the original code were flagged for manual review to verify correctness and address inconsistencies inherited from the source corpus. Terms that were not matched automatically were flagged for manual normalisation.

The construction of *expression_CIM* annotations involved combining individual entities into composite expressions representing full oncological concepts, such as the previous example “adénocarcinome canalaire du pancreas de grade 2”. Expressions meeting simple criteria (containing one morphology, and optionally one topography and/or one differentiation), were automatically reconstructed from their components and assigned a composite annotation. More complex cases, including expressionswith multiple entities per label, context dependent abbreviations, ambiguous phrasing or *expression_CIM* overlaps were flagged for manual and normalisation.

Special attention was given to expressions containing **“**adénopathi**-”** (e.g., *adénopathie*, *conglomérat adénopathique*, etc.). These cases often required a specific topography code (C77.-, referring to lymph nodes), but were context-dependent and not reliably annotated in the source data. To address this, expressions containing “adénopathi-“ were programmatically pre-processed by substituting the morphological component with a generic equivalent, “ganglion.” The resulting expression was then matched against the ICD-O-3 topographical dictionary. If a valid match was found, the associated C77.- topography code was applied. Expressions for which no valid match could be constructed were set aside for manual review.

This semi-automated workflow allowed for high-coverage, consistent ICD-O-3 normalisation at both the entity and expression level, balancing scalability with annotation quality. The result is a dataset enriched with structured oncology information suitable for downstream tasks in clinical NLP, semantic parsing, and ontology alignment.

### Correction of mistranslations

Because the synthetic clinical cases were translated from Spanish into French using the DeepL translation engine, some expressions were incorrectly rendered. This problem was particularly frequent with abbreviations and overly complex medical expressions, which were often left untranslated or were completely mistranslated, which was not representative of authentic French clinical language.

To address this, we implemented a systematic correction pipeline during the annotation process. First, annotators flagged mistranslated expressions when encountered in the corpus. These flagged strings were then used as seeds to automatically retrieve similar instances across the dataset. Each retrieved candidate was manually checked and flagged if necessary. In a second step, a Python script was developed to (i) retranslate flagged terms into appropriate French expressions, (ii) replace the incorrect spans directly in the text, and (iii) shift all existing annotations in the corresponding document to preserve alignment between the corrected text and the standoff annotation files.

This iterative process ensured that mistranslations were consistently corrected across the dataset. In total, 961 expressions were flagged and corrected, improving the linguistic quality and representativeness of the French corpus while maintaining annotation integrity.

### Annotation tools and workflow

Manual annotation was conducted using BRAT, a web-based interface supporting span-based entity annotation in the standoff.ann format. Annotators used brat to revise projected annotations from the original CANTEMIST corpus, add new entities (morphology, topography, differentiation, full expressions), and ensure precise alignment with the corresponding French texts.

To support the normalisation process, all annotated entities were automatically extracted from the BRAT.ann files into a structured CSV file, where each entry could be independently reviewed and matched to the appropriate ICD-O-3 code. This working format enabled efficient dictionary matching and allowed annotators to easily resolve inconsistencies and handle complex cases such as ambiguous or multi-entity expressions.

Annotators had access to the full original French text throughout the normalisation process. This allowed for context-sensitive interpretation of expressions. While many annotations could be assigned based on local lexical cues, certain expressions required broader contextual interpretation. For example, morphology expressions often carried implicit malignancy status (e.g., *adénome* vs. *adénocarcinome*), which was not always lexically specified. In some cases, context helped clarify whether a term referred to a *benign* (/0), *malignant* (/3), or *uncertain malignancy* (/1) process, particularly for more general or ambiguous terms such as *lésion*, *prolifération*, or even *tumeur*. Similarly, the interpretation of *primary* (/3), *secondary* (/6) or *uncertain* (/9) expressions depended on document-level cues. In contrast, differentiation grades (e.g., *grade 2*, *peu différencié*) were usually explicitly mentioned and required minimal contextual inference.

This reference to source context ensured that ICD-O-3 codes were not only structurally correct, but also semantically accurate in the clinical narrative.

A custom set of Python scripts, based on and extending the open-source bratly package, supported the annotation, normalisation and translation workflow. These tools automated the extraction and reintegration of annotations, ICD-O-3 dictionary matching, expression construction, and validation checks, as well as allowed integrative translation. Once the review process was complete, normalised codes were automatically propagated back into the.ann files, producing a consistent and fully enriched final dataset.

This combined methodology of manual annotation and programmatic tooling allowed for scalable, high-quality annotation across 1,301 documents and over 71,126 entities.

## Data Record

The dataset is available via the Zenodo repository at https://doi.org/10.5281/zenodo.17284817^18^. It comprises 1,301 synthetic oncological clinical texts in French, derived from the CANTEMIST corpus and translated through the FRASIMED initiative. The foundational CANTEMIST corpus and its translated FRASIMED counterpart are openly available under the Creative Commons Attribution 4.0 International (CC BY 4.0) license. The development, reuse, and open distribution of the FRACCO dataset fully comply with these original terms of use, and our dataset is made publicly available under the same CC BY 4.0 license. Each text file is provided in plain-text format (.txt) and is accompanied by annotation files (.ann) formatted according to the brat standoff annotation scheme. A.csv file of all annotations and ICD-O-3 normalisation is also available for processing and review.

A total of 1,301 annotation files is included, containing 71,126 manual annotations with ICD-O-3 normalisation. These span 399 distinct morphology codes, 273 topography codes, all 4 differentiation codes, and more than 2,000 unique combinations expressed as expression_CIM annotations (see Table 7 for detailed statistics). Annotations are separated into entity annotations and corresponding normalisation notes referencing ICD-O-3 terminology.

**Table 7:** Detailed Dataset Statistics.

Metric	Total	Min	Max	Mean	Median
Document Length (words)	1,031,844	186	2,352	793.12	752
Total Annotations	71,126	0	211	54.67	51
Entities Count by Type					
- *morphologie/expression_CIM*	25,634	0	68	19.70	18
- *topographie*	18,805	0	77	14.45	13
- *différenciation*	1’053	0	11	0.081	0
Annotation Span Length (words)					
- *morphologie*	—	1	21	1.52	1
- *topographie*	—	1	16	1.38	1
- *différenciation*	—	1	11	2.12	2
- *expression_CIM*	—	1	53	3.72	2

All files are stored in a flat directory structure. Each.txt file shares its filename prefix with its associated.ann file to denote correspondence (e.g., cc_onco859.txt, cc_onco859.ann). These filename prefixes correspond directly to the original CANTEMIST and FRASIMED corpus identifiers, enabling users to trace back and recover the source Spanish texts if needed for comparative or contextual analysis.

## Technical Validation

To ensure the reliability and consistency of the dataset, we performed structured validation procedures across both the span annotation and ICD-O-3 code normalisation phases. These two layers of validation reflect the dual nature of the annotation task: identifying relevant medical expressions in the text and assigning them appropriate standardised codes. In addition, we evaluated the dataset through NER model fine-tuning to confirm that the annotation scheme is coherent, the labels are learnable by modern architectures, and the corpus is of sufficient size to support supervised training. Together, these procedures confirm both the internal quality of the annotations and the usability of the dataset for NLP applications.

### Span Annotation Validation

The first stage involved validating text span annotations, highlighting expressions related to morphology, topography, and differentiation, across all 1,301 French clinical texts. Two biomedical expert annotators worked independently using a shared guideline that emerged from an initial calibration phase. During annotation, inconsistencies were noted particularly in non-pre-annotated terms, those not projected from the original CANTEMIST/FRASIMED dataset, which were harder to detect. To mitigate this, we implemented an automatic flagging mechanism that identified recurrent untagged terms, surfacing them in subsequent rounds. This significantly reduced cases where an annotation was completely missed by one annotator.

In the first round of full annotation, inter-annotator agreement (IAA) was computed using both **Hard** (exact span match) and **Soft** (partial overlap) criteria. As summarized in Tables 8 and 9, soft F1 scores ranged from 0.82 to 0.92, while hard F1 scores ranged from 0.70 to 0.78 across categories, with the exception of one outlier category (*differenciation*) which scored 0.48. Error analysis showed that most disagreements were due to span boundary differences: one annotator would often include more contextual information, while the other opted for a more concise span. The particularly low score for *differenciation* annotations was traced to a systematic discrepancy in span selection, one annotator consistently annotated expressions like “de grade 2,” while the other selected only “grade 2,” leading to a technical mismatch despite semantic equivalence.

**Table 8:** Inter-annotator agreement using “soft” matching (partial overlap considered true).

Label	n	Precision (A in B)	Recall (B in A)	F-1 score
*expression_CIM*	26,297	0.955	0.759	0.846
*morphologie*	26,539	0.958	0.782	0.861
*topographie*	19,712	0.838	0.811	0.824
*differenciation*	1,137	0.911	0.923	0.917

**Table 9:** Inter-annotator agreement using “hard” matching (only perfect overlap considered true).

Label	n	Precision (A in B)	Recall (B in A)	F-1 score
*expression_CIM*	29,599	0.792	0.629	0.701
*morphologie*	28,326	0.873	0.712	0.784
*topographie*	21,032	0.758	0.733	0.745
*differenciation*	1,592	0.481	0.487	0.484

Across other categories, disagreements were largely attributed to different interpretations of what textual information was relevant to include, rather than divergent understandings of the clinical meaning. To produce the final gold-standard corpus, we implemented a structured reconciliation phase. Each expert was provided with the annotations of the other for cross-validation; partial matches and missing entities were reviewed by both parties to establish common ground. Remaining unresolved discrepancies were adjudicated by a third expert who served as the final authority. Unresolved items defaulted to the version provided by this third expert, ensuring a harmonised final entity set.

For the final reconciliation phase, each annotator reviewed all partial-match cases. Annotations missing completely from one or the other annotator were reviewed and validated by a third annotator. Disagreements were resolved by consensus, and agreed annotations were included in the harmonised corpus; unresolved items default to the version provided by one annotator. This process yielded improved agreement, and a final, high-quality entity annotation set.

### ICD-O-3 Normalisation Validation

For normalisation, annotated spans were exported into a structured CSV and matched via exact string matching against the ICD-O-3 terminology dictionary. This method successfully normalised approximately 55,000 annotations (≈78%) automatically. The remaining ~15,000 complex expressions were manually reviewed. To evaluate both pathways, we conducted a stratified validation calculated to yield 95% confidence and 3% margin. From this, two validation corpora were extracted, an “automatic” and a “manual” corpus of respectively 1,060 and 1,000 expressions. As stated in the corresponding section, these subsets represent distinct levels of complexity; the automatic corpus primarily comprises high-certainty, exact string matches, whereas the manual corpus is composed exclusively of complex expressions that failed initial dictionary matching. Each corpus was then reviewed by a different expert, for codes to be validated.

In validating the automatic subset, the inter-annotator agreement (IAA) on the sampled 1,060 reached an 80.65% concordance. This discrepancy is expected, as the manual subset serves as a repository for all complex and semantically ambiguous expressions that require expert interpretation beyond simple lexicon lookups. This underscores that the difficulty lies not in the workflow, but in the inherent ambiguity of complex clinical phrasing.

Error analysis revealed several common sources of mismatch:

- Ontology limitations: ICD-O-3 sometimes lacked precise codes for composite expressions, leading to multiple potential mappings, consistent with known challenges in ontology mapping^20–22^.
- Semantic ambiguity: Different interpretations of clinical meaning led to variant code assignment decisions between annotators.
- Complex phrasing: Certain expressions combined elements in ways not directly supported by ICD-O-3, requiring consensus interpretation.

Annotators discussed all such edge cases, reached consensus coding, and propagated the agreed-upon mapping across the full dataset. A full list of the reviewed edge cases can be found in supplementary material.

Together, these validation steps demonstrate that our normalisation workflow, combining automated dictionary matching, expert review, and targeted error resolution, yields a high-quality annotated corpus suitable for downstream biomedical NLP applications.

### NER model fine-tuning on FRACCO

To demonstrate how this dataset can be leveraged for machine learning applications, we fine-tuned several pretrained named entity recognition (NER) models on the annotated texts. The goal was not to optimise model performance, but to provide first results to establish benchmark scores that enable comparison across future studies. Those results illustrate how different BERT-based models with varying pretraining corpora and scopes perform on the dataset.

The corpus, comprising 1,301 clinical case texts, a dataset size comparable to other French biomedical NER benchmarks^23^, was divided into a training set (80%) and a held-out test set (20%). The latter was kept fixed across all experiments and used only for final evaluation. Within the training portion, a validation set corresponding to 10% of the training data (8% of the full corpus) was resampled at each run, ensuring that training/validation splits varied while the test set remained constant. This design reduces dependence on any single validation partition while maintaining comparability. We fine-tuned five different BERT-based models, each trained with five fixed initialisation seeds.

**Fig. 4:**
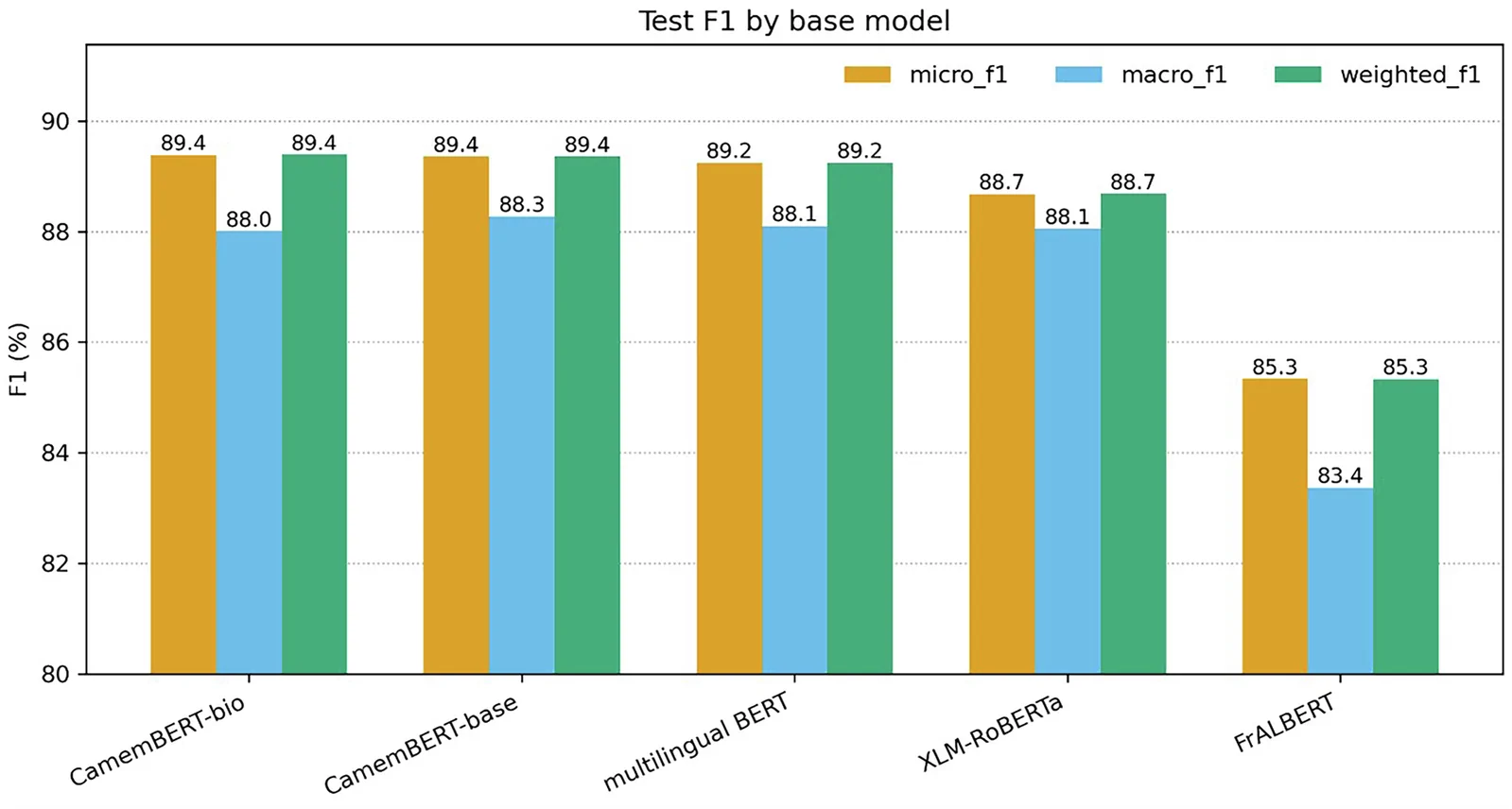
Overall predictive performance of the evaluated fine-tuned models. Bar chart comparing test set F1 scores across five base models. Coloured bars indicate the specific evaluation metric used (orange: micro F1, light blue: macro F1, green: weighted F1).

**Fig. 5:**
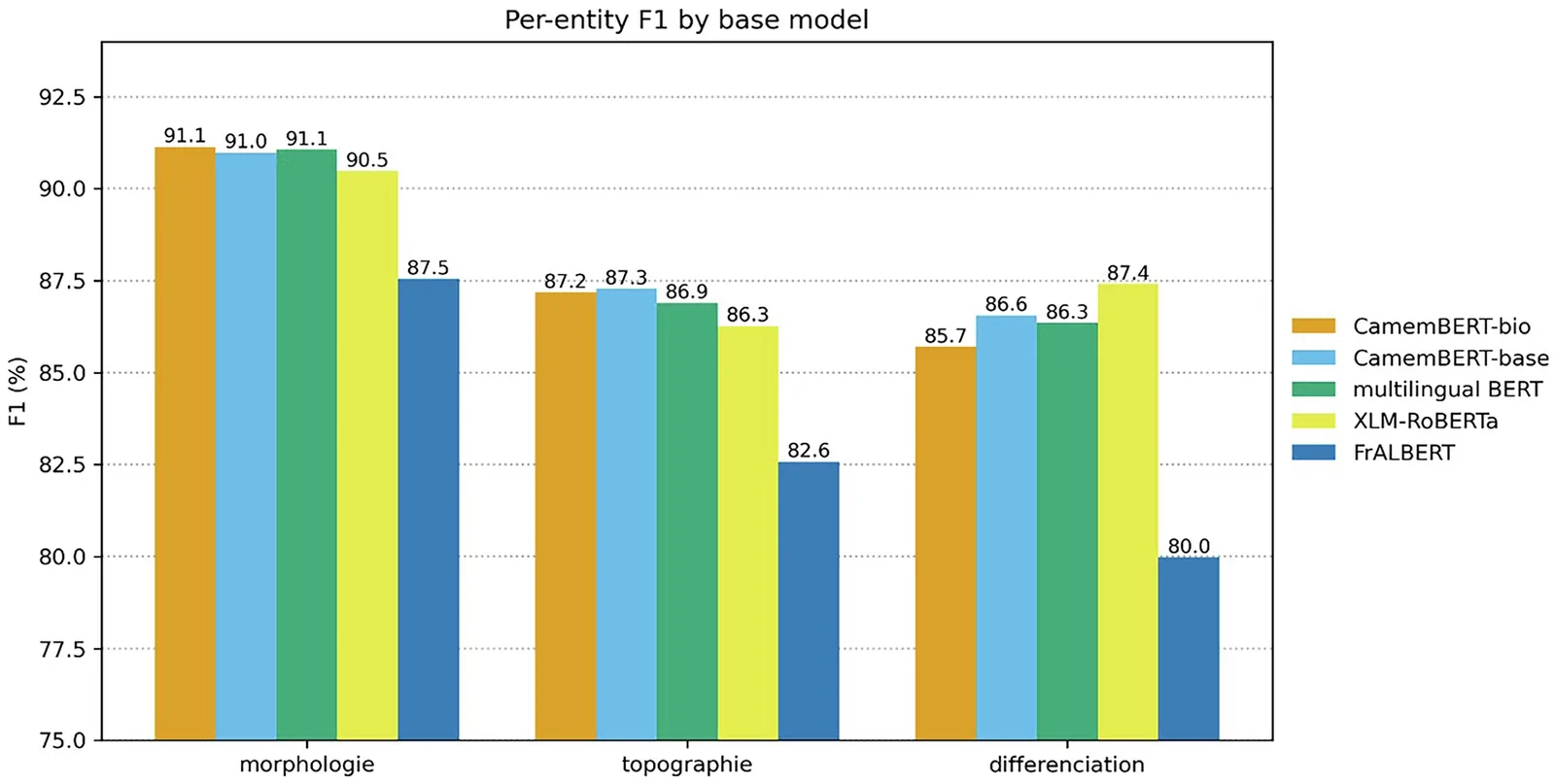
Predictive performance of fine-tuned models by entity type. Bar chart detailing F1 scores for the extraction of morphology, topography, and differentiation entities. Coloured bars denote the specific base model evaluated.

Performance, averaged across seeds and evaluated with exact span and label matching, is summarised in Figs. 4 and 5. Four of the five BERT-based models performed comparably, despite varying pretraining strategies. CamemBERT-base (pretrained on general-domain French texts drawn from the OSCAR Common Crawl corpus and French Wikipedia) and CamemBERT-bio^24^ (a continued-pretraining adaptation of CamemBERT-base on French biomedical corpora) both reached 89.4% weighted-F1. The multilingual general-domain models scored marginally lower, with multilingual BERT reaching 89.2% weighted-F1 and XLM-RoBERTa reaching 88.7%. By contrast, frALBERT underperformed at 85.3% weighted-F1, consistent with its smaller size, more limited pretraining (~4 GB of French Wikipedia), and efficiency-oriented design^25^.

Differences among the four larger models were ≤ 1 F1 point, comparable to variation expected across random seeds. This contrasts with evaluations of French biomedical NER, where domain-adapted models such as CamemBERT-bio typically gain 2-3 F1 points over general-domain CamemBERT^23,26^ and monolingual French encoders usually outperform multilingual BERT^27^. In our case, no meaningful differences between model types were observed. This is likely explained by the fact that oncological terminology comprises frequent morphology and topography terms that are already well represented in general corpora such as OSCAR and Wikipedia, so additional biomedical adaptation contributes less than in tasks where vocabulary is rarer. The advantage of monolingual pretraining is also reduced in this setting, as the corpus provides a high density of short, repetitive entity mentions that can be effectively learned by both general-domain and multilingual models. Overall, the convergence of the larger models indicates that the dataset yields stable results across different pretrained variants.

At the entity level, *morphologie* achieved the highest F1 scores across models (90.5–91.1% for the larger models). *Topographie* and *différenciation* performed at comparable levels, with F1 scores ranging from 86.3–87.2% and 85.7–87.4%, respectively, despite the much smaller number of *différenciation* annotations. This is explained by the greater reproducibility of grade expressions, which are short and lexically homogeneous (e.g. “*de grade I*”). In contrast, *topographie* covers a wide lexical and semantic space. This is consistent with the ICD-O-3 structure, which defines only four differentiation codes compared to nearly 250 topography codes. Despite the strong imbalance across categories—*morphologie* and *topographie* together account for over 95% of annotations—F1 scores remained stable across all labels. This indicates that class imbalance did not hinder learnability, particularly for *différenciation*, which achieved high performance despite representing only ~2% of the data.

## Usage Notes

The FRACCO dataset is designed to support the development and evaluation of clinical natural language processing (NLP) systems in French, specifically within the oncology domain. Primary downstream applications include training and benchmarking machine learning models for named entity recognition (NER), concept normalisation, and automated clinical coding.

Because the document identifiers in FRACCO exactly match those in the original CANTEMIST corpus, researchers can seamlessly cross-reference the French clinical cases with their Spanish-language counterparts. This parallel structure makes the dataset suitable for researchers conducting multilingual or cross-lingual text-mining experiments.

While no additional preprocessing is required to access the raw text and standard BRAT annotations, researchers performing large-scale analyses or training deep learning pipelines may need to convert the data into alternative formats (e.g., CSV or JSON). Users are encouraged to utilise the custom Python scripts (detailed in the *Code Availability* section) to easily parse the annotations, filter expressions by specific ICD-O-3 codes or adapt the data to their preferred text annotation schemas.

## Supplementary information

Supplementary information

Supplementary information

Supplementary information

Supplementary information

## Data Availability

The FRACCO dataset is archived and publicly available on Zenodo to ensure long-term accessibility and reproducibility: https://doi.org/10.5281/zenodo.17284817. The dataset contains pairs of.ann and.txt files, to be used with BRAT (brat rapid annotation tool) (https://brat.nlplab.org/) or any compatible software.

## Code availability

All code used to process, validate, and explore the dataset is available at a dedicated GitHub repository: https://github.com/SimedDataTeam/FRACCO. This includes tools for processing BRAT or CSV-formatted annotations. All scripts are detailed in the repository “readme.md” file.
